# Strategies for lung- and diaphragm-protective ventilation in acute hypoxemic respiratory failure: a physiological trial

**DOI:** 10.1186/s13054-022-04123-9

**Published:** 2022-08-29

**Authors:** Jose Dianti, Samira Fard, Jenna Wong, Timothy C. Y. Chan, Lorenzo Del Sorbo, Eddy Fan, Marcelo B. Passos Amato, John Granton, Lisa Burry, W. Darlene Reid, Binghao Zhang, Damian Ratano, Shaf Keshavjee, Arthur S. Slutsky, Laurent J. Brochard, Niall D. Ferguson, Ewan C. Goligher

**Affiliations:** 1 Interdepartmental Division of Critical Care Medicine, University of Toronto, Toronto, Canada; 2 Division of Respirology, Department of Medicine, University Health Network, Toronto, Canada; 3 Department of Respiratory Therapy, University Health Network, Toronto, Canada; 4 Department of Mechanical and Industrial Engineering, University of Toronto, Toronto, Canada; 5 Heart Institute (Incor), Hospital das Clínicas da Faculdade de Medicina da Universidade de São Paulo, São Paulo, Brazil; 6 Department of Pharmacy, Mount Sinai Hospital, Toronto, Canada; 7 Leslie Dan Faculty of Pharmacy, University of Toronto, Toronto, Canada; 8 Department of Physical Therapy, University of Toronto, Toronto, Canada; 9 Department of Surgery, University of Toronto, Toronto, Toronto Canada; 10 Keenan Centre for Biomedical Research, Li Ka Shing Knowledge Institute, St. Michael’s Hospital, Toronto, Canada; 11 Toronto General Hospital Research Institute, 9-MaRS-9024, 585 University Avenue, Toronto, ON M5G 2N2 Canada; 12 Institute for Health Policy, Management, and Evaluation, University of Toronto, Toronto, Canada; 13 Department of Physiology, University of Toronto, Toronto, Canada

**Keywords:** Hypoxemic respiratory failure, Lung-protective ventilation, Diaphragm-protective ventilation, Mechanical ventilation

## Abstract

**Background:**

Insufficient or excessive respiratory effort during acute hypoxemic respiratory failure (AHRF) increases the risk of lung and diaphragm injury. We sought to establish whether respiratory effort can be optimized to achieve lung- and diaphragm-protective (LDP) targets (esophageal pressure swing − 3 to − 8 cm H_2_O; dynamic transpulmonary driving pressure ≤ 15 cm H_2_O) during AHRF.

**Methods:**

In patients with early AHRF, spontaneous breathing was initiated as soon as passive ventilation was not deemed mandatory. Inspiratory pressure, sedation, positive end-expiratory pressure (PEEP), and sweep gas flow (in patients receiving veno-venous extracorporeal membrane oxygenation (VV-ECMO)) were systematically titrated to achieve LDP targets. Additionally, partial neuromuscular blockade (pNMBA) was administered in patients with refractory excessive respiratory effort.

**Results:**

Of 30 patients enrolled, most had severe AHRF; 16 required VV-ECMO. Respiratory effort was absent in all at enrolment. After initiating spontaneous breathing, most exhibited high respiratory effort and only 6/30 met LDP targets. After titrating ventilation, sedation, and sweep gas flow, LDP targets were achieved in 20/30. LDP targets were more likely to be achieved in patients on VV-ECMO (median OR 10, 95% CrI 2, 81) and at the PEEP level associated with improved dynamic compliance (median OR 33, 95% CrI 5, 898). Administration of pNMBA to patients with refractory excessive effort was well-tolerated and effectively achieved LDP targets.

**Conclusion:**

Respiratory effort is frequently absent  under deep sedation but becomes excessive when spontaneous breathing is permitted in patients with moderate or severe AHRF. Systematically titrating ventilation and sedation can optimize respiratory effort for lung and diaphragm protection in most patients. VV-ECMO can greatly facilitate the delivery of a LDP strategy.

*Trial registration*: This trial was registered in Clinicaltrials.gov in August 2018 (NCT03612583).

**Supplementary Information:**

The online version contains supplementary material available at 10.1186/s13054-022-04123-9.

## Background

Patients with acute hypoxemic respiratory failure (AHRF) commonly require mechanical ventilation. Although potentially lifesaving, complications of mechanical ventilation such as ventilator-induced diaphragm dysfunction can adversely affect patient outcomes [1–3]. Excessive ventilatory support and heavy sedation can suppress respiratory effort and cause disuse atrophy of the diaphragm [4]. Restoring respiratory effort may prevent diaphragm atrophy and weakness and improve outcomes [5]. These patients, however, often have excessive respiratory efforts [6], which may result in injurious lung stress and strain (patient self-inflicted lung injury) and load-induced diaphragm injury (myotrauma) [5, 7].

To avoid these injuries, a lung- and diaphragm-protective (LDP) approach has been proposed. This approach specifies putatively protective ranges for both respiratory effort and lung-distending pressure during mechanical ventilation. A recent trial demonstrated that titrating inspiratory support to achieve diaphragm-protective targets for respiratory effort increased the probability of achieving appropriate effort levels in patients who were already on assisted ventilation for several days [8]. Early intervention is crucial, however, because the risk of injury to the lung and diaphragm is likely highest during the early course of ventilation. It remains unknown whether LDP targets can be achieved during AHRF prior to the weaning phase. Previous studies suggest that higher positive end-expiratory pressure (PEEP) [9], extracorporeal CO_2_ removal, and partial neuromuscular blockade may be useful to control respiratory effort [10]. We conducted a physiological trial in patients with early AHRF to establish whether LDP targets could be achieved by systematically titrating inspiratory pressure, sedation, positive end-expiratory pressure and, in patients already on veno-venous extracorporeal membrane oxygenation (VV-ECMO), sweep gas flow. In patients refractory to these interventions, we evaluated whether LDP targets could be achieved by applying adjunctive partial neuromuscular blockade.

## Methods

This was a physiological randomized cross-over clinical trial testing the effect of different interventions on lung-distending pressure (quantified by the dynamic transpulmonary driving pressure, ∆P_L,dyn_) and respiratory effort (quantified by the esophageal pressure swing, ∆Pes) to achieve LDP targets in patients with AHRF (Trial registration: NCT03612583, University Health Network Research Ethics Board #18-5644). The trial was initially designed to ascertain whether LDP targets could be achieved and maintained over 24 h. Owing to the complexity of the trial intervention and the focus on physiological effects, this approach was modified to focus on whether LDP targets could be achieved by the end of trial intervention.

### Study population and setting

This trial was conducted in the medical-surgical intensive care unit (ICU) at the Toronto General Hospital, a quaternary academic hospital serving as the major provincial referral centre for patients with severe acute hypoxemic respiratory failure. We enrolled invasively mechanically ventilated patients with AHRF (including patients receiving veno-venous extracorporeal membrane oxygenation, VV-ECMO), defined by PaO_2_:FiO_2_ ≤ 300 mm Hg not attributable to cardiogenic pulmonary edema at enrolment or before cannulation for VV-ECMO. Patients were excluded if they had any contraindication to esophageal catheterization, acute brain injury or intracranial hypertension, or were expected to be liberated from mechanical ventilation within 24 h. Eligible patients were identified by daily screening and enrolled as early as possible following initiation of invasive ventilation.

### Measurements

Airway pressure (Paw), flow, and tidal volume (V_T_) were measured by a pneumotachograph at the airway opening (FluxMed, MBMed, Buenos Aires, Argentina). An esophageal balloon catheter (NutriVent, Modena, Italy) was placed to measure ∆Pes and ∆P_L,dyn_. Diaphragm electrical activity (Edi) was measured by a specially fitted esophageal catheter connected to the Servo-U ventilator (Getinge, Solna, Sweden).

Transpulmonary pressure (*P*_L_) was computed by real-time subtraction of Pes from Paw. Static airway driving pressure (∆Paw) and transpulmonary driving pressure (∆*P*_L_) were measured by applying a transient end-inspiratory hold and a transient end-expiratory hold on the ventilator to measure plateau airway pressure (Pplat)  plateau esophageal pressure (Pes,ei), total PEEP (PEEP_tot_), and end-expiratory esophageal pressure (Pes,ee), respectively. ∆Pes was computed as the difference between end-expiratory Pes and the nadir of inspiratory Pes. ∆P_L,dyn_ was computed as the inspiratory swing in transpulmonary pressure (peak *P*_L_ – end-expiratory *P*_L_).

Dynamic lung compliance (*C*_L,dyn_) was computed as:

$$C_{{\text{L,dyn}}} = \frac{{V_{{\text{T}}} }}{{\Delta P_{{\text{L,dyn}}} }}$$

### Study protocol

**Fig. 1:**
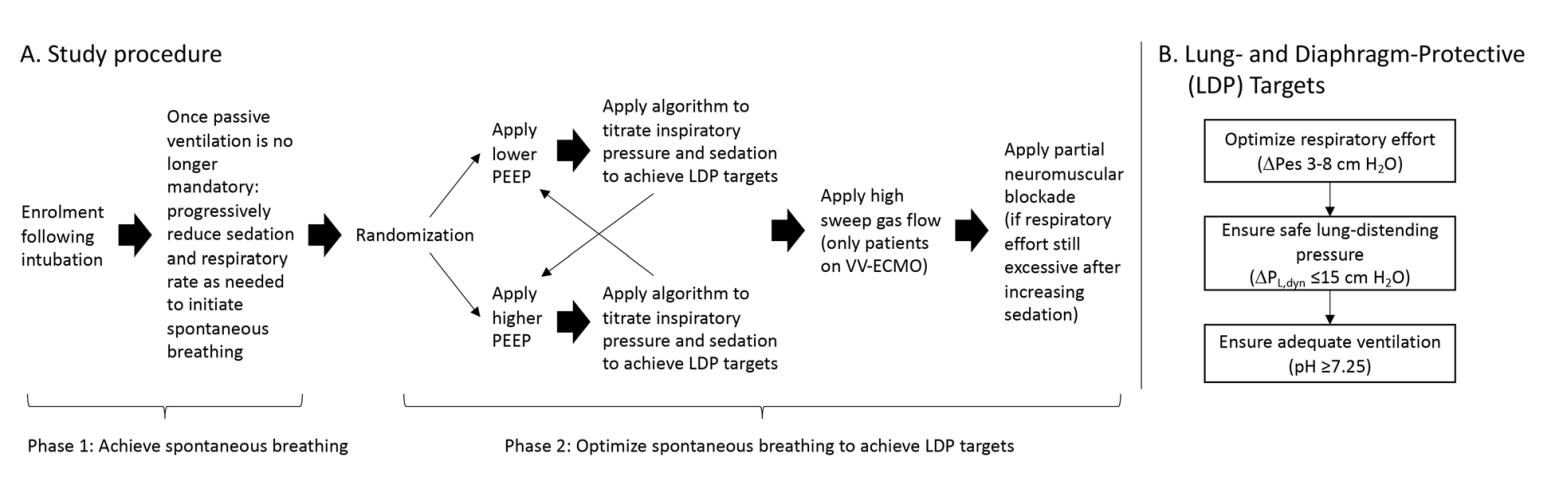
Trial design. **A** Study procedure to test the effect of different interventions on the probability of achieving lung- and diaphragm-protective targets. **B** Approach to adjusting ventilation and sedation to achieve lung- and diaphragm-protective targets. The algorithm used for titration in the trial is provided in Additional file 1: Fig. S2. PEEP: positive end-expiratory pressure; VV-ECMO: veno-venous extracorporeal membrane oxygenation; LDP: lung- and diaphragm-protective; ∆Pes: esophageal pressure swing; ∆*P*_L,dyn_: dynamic driving transpulmonary pressure

The protocol proceeded in two phases (Fig. 1A). The goal of the first phase was to initiate spontaneous breathing. This was accomplished by progressively reducing sedation, respiratory rate set on the ventilator, and sweep gas flow (in patients on VV-ECMO) according to a standardized procedure (Additional file 1: Fig. S1) until patients were continuously triggering the ventilator. During this phase, ventilator mode and settings were otherwise managed by the clinician in charge of the patient following usual clinical practice. Once spontaneous respiratory efforts were present, the ventilator mode was converted to pressure support ventilation mode and adjusted per standard clinical procedure to achieve respiratory frequency ≤ 35 breaths per minute and V_T_ of ≤ 8 cc/kg predicted body weight, or pressure assist-control mode with inspiratory pressure and time titrated to achieve ≤ 8 cc/kg predicted body weight and set rate adjusted to maintain patient triggered breaths and respiratory frequency ≤ 35.

The goal of the second phase was to test whether spontaneous breathing could be modulated to achieved LDP targets (∆Pes −3 to −8 cm H_2_O and ∆*P*_L,dyn_ ≤ 15 cm H_2_O (Fig. 1B). The rationale for these targets is detailed in Additional file 1: Study Protocol, and elsewhere [5]. To compare the effects of higher and lower PEEP levels on LDP targets, patients were randomly assigned to lower PEEP (PEEP of 8 cm H_2_O or the lowest PEEP level required to maintain FiO_2_ ≤ 90% and SpO_2_ ≥ 90%) or higher PEEP (PEEP sufficient to maintain end-expiratory *P*_L_ between 2–3 cm H_2_O). Inspiratory pressure and the propofol infusion rate were then adjusted according to a predefined stepwise algorithm (Additional file 1: Fig. S2) to attempt to achieve LDP targets. This titration procedure was then repeated after patients were crossed over to the alternate PEEP level. In patients on VV-ECMO, sweep gas flow was then increased to the maximum level that did not induce apnea and the titration procedure was repeated again. Finally, in patients whose respiratory effort remained excessive after all prior interventions including maximal sedation with propofol, partial neuromuscular blockade was applied using intermittent boluses (0.5–1 mg) of cisatracurium. The protocol is described in detail in Additional file 1: Study Protocol.

### Outcomes and statistical analysis

The proportion of patients meeting LDP targets at the end of each step in the trial was computed with 95% confidence intervals. The effect of each studied intervention on the probability of meeting LDP targets was quantified using a Bayesian generalized mixed effects model with non-informative priors [11]. For this model, we report the median and 95% credible intervals (CrI) for the odds ratio (OR) and the posterior probability of OR > 1 for each intervention. Because VV-ECMO was found to significantly modify the effect of LDP titration, the results are also reported separately for patients receiving or not receiving VV-ECMO.

The effects of each study intervention on ∆Pes and ∆P_L,dyn_ were quantified by linear mixed effects models. Since PEEP is known to have highly variable effects on lung mechanics, the probability of meeting LDP targets was also compared between the two PEEP levels classified by whether dynamic lung compliance was improved or worsened by that PEEP level in comparison to the alternate PEEP level. Physiological and clinical characteristics were compared between patients in whom LDP targets were achieved or not achieved using the standardized mean difference.

The original planned sample size for the trial was 40 patients. Owing to unexpectedly slow recruitment during the 27-month study period (partly due to interruptions from the COVID-19 pandemic), we reviewed the sample size calculation and decided that a sample size of 30 subjects was sufficient to estimate the proportion of patients in whom LDP targets were achieved after PEEP and sweep gas flow titrations were completed with acceptable precision (confidence intervals of ± 16% with 30 patients vs. confidence intervals ± 12% with 40 patients). This decision was made prior to any data analysis.

A detailed description of additional methods is provided in Additional file 1: Details on the Statistical Analysis. Statistical analyses were performed using R v4.2.0 (R Foundation for Statistical Computing, https://www.R-project.org). Bayesian models were built using the {rstanarm} package [12].

## Results

Between January 2019 and March 2021, 134 patients were assessed for eligibility; 35 were consented and enrolled. Of these, 1 patient was excluded after self-extubation, 2 patients were transferred back to the referring hospital before starting the protocol, and 2 patients died before the LDP titration procedure could be initiated (Additional file 1: Fig. S3). Thirty patients underwent the LDP titration procedure and were included in the final analysis. Baseline clinical and physiological characteristics are reported in Table 1.

**Table 1:** Patient characteristics and ventilation variables at enrolment

Age, median (IQR)	54 (49, 60)
Female sex, *n* (%)	10 (33)
APACHE II, median (IQR)	21 (18, 24)
SOFA, median (IQR)	11 (10, 12)
AHRF severity, median (IQR)	
Moderate: PaO_2_:FiO_2_ 100-200 mm Hg	7 (23)
Severe: PaO_2_:FiO_2_ <100 mm Hg	23 (77)
Comorbidities, *n* (%)	
COPD	5 (17)
Asthma	3 (10)
Diabetes	8 (27)
Chronic kidney disease	1 (3)
Interstitial lung disease	7 (23)
Etiology of respiratory failure	
Bacterial pneumonia	13 (43)
Fungal pneumonia	1 (3)
Viral pneumonia (non-COVID-19)	2 (7)
COVID-19 pneumonia	14 (47)
SAS, median (IQR)	1 (1,2)
PaO_2_:FiO_2_ (mm Hg)*, median (IQR)	109 (79, 167)
Ventilatory ratio†, median (IQR)	2 (1.6, 2.7)
Mode of ventilation, *n* (%)	
Assist-control volume ventilation	21 (70)
Assist-control pressure ventilation	9 (30)
V_T_/PBW (ml/kg), median (IQR)	6 (5, 8)
PEEP (cm H_2_O), median (IQR)	10 (9, 14)
Driving pressure (cm H_2_O), median (IQR)	17 (12, 19)
Respiratory system compliance (ml/cm H_2_O), median (IQR)	23 (16, 37)
Normalized respiratory system elastance (cm H2O/ml/PBW), median (IQR)	2.6 (1.9, 5)
ECLS blood flow, (L/min) median (IQR)	5 (4, 5)
Sweep gas flow (L/min), median (IQR)	5 (4, 5)

*IQR* interquartile range, *COPD* chronic obstructive pulmonary disease, *SOFA* sequential organ failure assessment, *SAS* sedation-agitation score, *V*_*T*_ tidal volume, *PEEP* positive end-expiratory pressure, *PBW* predicted body weight, *ECLS* extracorporeal life support

*Represents only patients not receiving VV-ECMO (*n* = 14). Values of PaO_2_:FiO_2_ may not be representative of lung function in patients receiving VV-ECMO at enrolment

^†^Represents only patients not receiving VV-ECMO (*n* = 14). Values of VR may not be representative of lung function in patients receiving VV-ECMO at enrolment

### Phase 1—Initiating spontaneous breathing

**Fig. 2:**
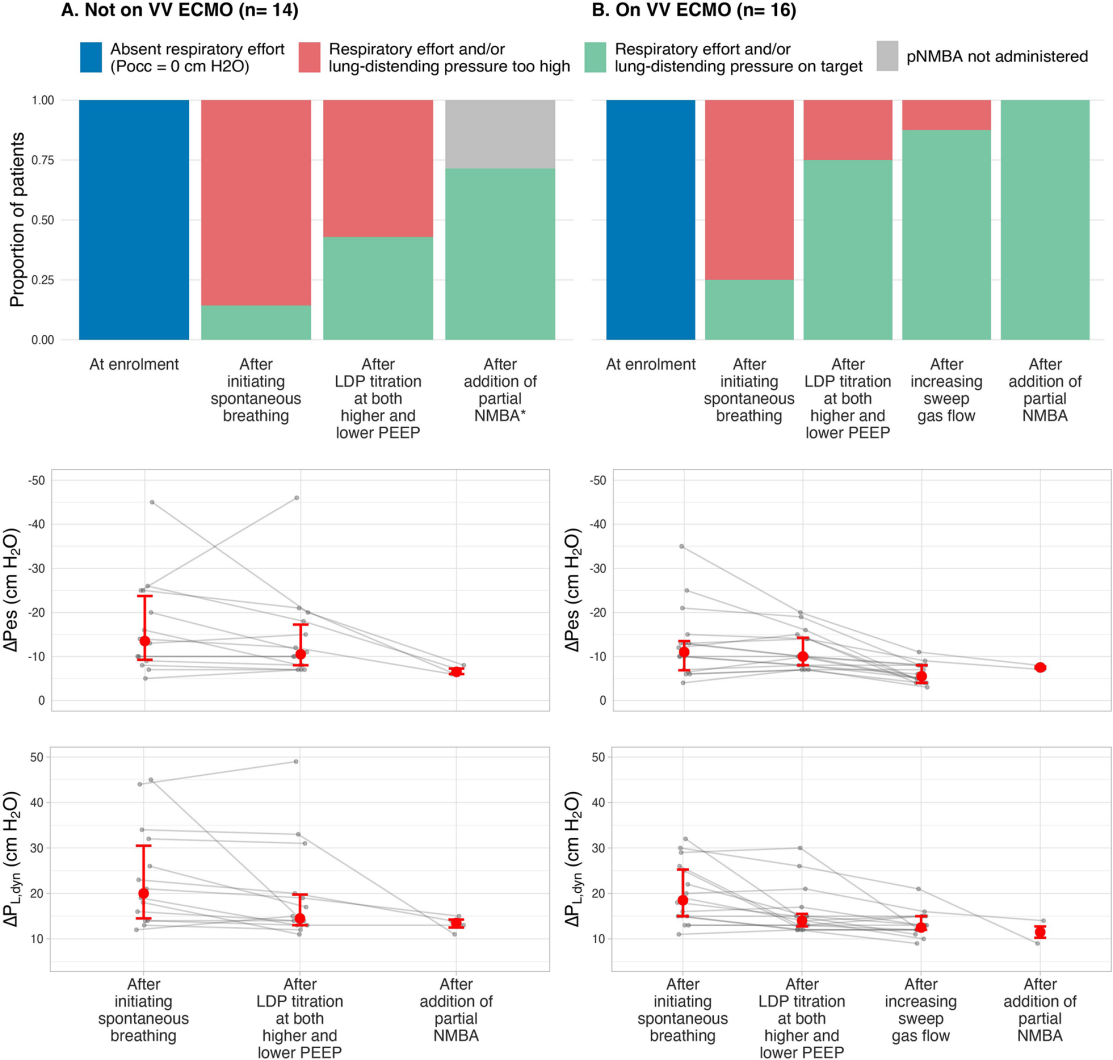
Physiological outcomes after each step in the trial. The proportion of patients who met lung and diaphragm-protective (LDP) targets at the end of each study phase in those not receiving VV-ECMO (**A**) and those receiving VV-ECMO (**B**). Below the stacked bar plots, the corresponding distributions of respiratory effort (ΔPes) and lung-distending pressure (ΔP_L,dyn_) are shown. Error bars represent 25^th^ and 75^th^ percentiles with median (circle). *Not all eligible patients received partial neuromuscular blockade due to decision of the attending physician. LDP: lung and diaphragm protection, VV ECMO: veno-venous extracorporeal membrane oxygenation, pNMBA: partial neuromuscular blockade, ∆Pes: esophageal pressure swing, ∆P_L,dyn_: dynamic transpulmonary driving pressure

At enrolment, 0/30 patients (0%, 95% CI 0, 11%) met LDP targets. In all cases this was due to absent or insufficient respiratory effort (Fig. 2). Median (IQR) static driving pressure at enrolment was 16 cm H_2_O (12, 18 cm H_2_O).

After reducing sedation, set respiratory rate on the ventilator, and sweep gas flow to initiate patient spontaneous breathing (and prior to applying the LDP titration algorithm), most patients exhibited excessive ∆Pes and ∆*P*_L,dyn_ (Fig. 2) and a minority (6/30, 20%, 95% CI 10, 37%) met LDP targets. Median time from enrolment to achieving spontaneous breathing was 1 day (IQR 1, 4 days).

### Phase 2—Optimizing spontaneous breathing: titrating ventilation and sedation

Respiratory mechanics, gas exchange, ∆Pes, and ∆P_L,dyn_ before and after LDP titration are reported in Fig. 2 and Additional file 1: Table S1. Changes in ventilation and sedation based on the algorithm are described in Additional file 1: Changes in Ventilation and Sedation During the LDP Titration Procedure and in Additional file 1: Fig. S4. Most patients (27/30) were ventilated in pressure support ventilation mode. The remaining 3 patients were ventilated in assist-control pressure control mode.

Overall, 18/30 patients met LDP targets after completing the titration procedure at both higher and lower PEEP levels (in comparison to end of phase 1: median OR 37, 95% CrI 4, 992). LDP targets were achieved in 6/14 patients not receiving VV-ECMO (in comparison to end of phase 1: median OR 12, 95% CrI 1, 545) and in 12/16 patients receiving VV-ECMO (in comparison to end of phase 1: median OR 33, 95% CrI 3, 1635) (Fig. 2). Among patients with excessive respiratory effort (∆Pes < –8 cm H_2_O) at the end of phase 1, the LDP titration procedure attenuated ∆Pes (mean difference 3 cm H_2_O, 95% CrI –1, 7 cm H_2_O) and ∆P_L,dyn_ (mean difference –4 cm H_2_O, 95% CrI –7, 1 cm H_2_O) (Fig. 2). Physiological and clinical characteristics of patients who did or did not meet LDP targets after completing the LDP titration procedure at both PEEP levels are shown in Additional file 1: Table S2. A higher fentanyl infusion rate was associated with successfully achieving LDP targets (Additional file 1: Fig. S4).

In a sensitivity analysis specifying more liberal LDP targets (∆Pes −3 to −12 cm H_2_O, ∆P_L,dyn_ ≤ 20 cm H_2_O), 22/30 patients met LDP targets after LDP titration at both PEEP levels (73%, 95% CI 56, 86%), including 9/14 patients not receiving VV-ECMO (64%, 95% CI 46, 79%) and 13/16 patients receiving VV-ECMO (81%, 95% CI 64, 91%).

The LDP titration procedure was generally well tolerated. The most commonly observed adverse event associated with the intervention at any time point was respiratory acidosis in 5 (16%) patients. The protocol could not be completed in 1 patient due to a significant increase in the work of breathing following application of lower PEEP (∆Pes < –25 cm H_2_O and signs of respiratory distress). Two (6%) patients developed transient hypotension requiring an increased vasopressor dose due to increases in sedation. No severe patient-ventilator dyssynchrony was observed.

### Phase 2—Optimizing spontaneous breathing: specific effect of PEEP titration

**Fig. 3:**
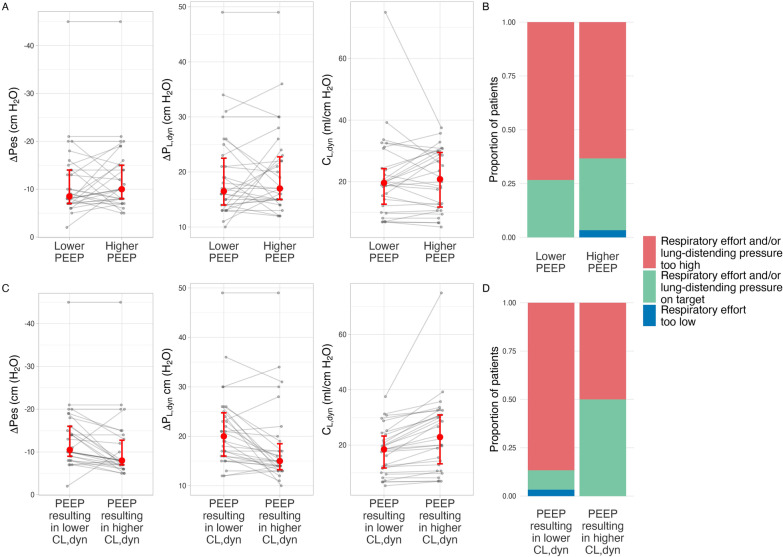
Effect of modifying PEEP on respiratory effort and lung-distending pressure. There was no difference in ∆Pes, ∆P_L,dyn_, or the probability of meeting lung- and diaphragm-protective targets between higher or lower PEEP levels (**A**, **B**). The effects of higher vs. lower PEEP on ΔPes and ΔP_L,dyn_, varied widely between patients according to the effect of changing PEEP on dynamic lung compliance (**C**). The probability of meeting LDP targets at the PEEP level associated with higher dynamic lung compliance was greater in comparison to the PEEP level associated with lower dynamic lung compliance (**D**). Error bars represent 25^th^ and 75^th^ percentiles with median (circle). LDP: lung and diaphragm protection, ∆Pes: esophageal pressure swing, ∆P_L,dyn_: dynamic transpulmonary driving pressure, C_L,dyn_: dynamic lung compliance, PEEP: positive end-expiratory pressure

Median (IQR) PEEP at the higher PEEP step was 14 cm H_2_O (13, 16 cm H_2_O); at the lower PEEP step it was 8 cm H_2_O (8, 8 cm H_2_O). Higher PEEP had, on average, minimal effect on ∆Pes (mean difference –1 cm H_2_O, 95% CrI –3, 1 cm H_2_O) or ∆P_L,dyn_ (mean difference −1 cm H_2_O, 95% CrI −3, 2 cm H_2_O) (Fig. 3A). The probability of meeting LDP targets was similar at higher and lower PEEP levels (47% vs. 40%, median OR 1.5, 95% CrI 0.5, 5) (Fig. 3B).

However, the effect of PEEP on ∆Pes and ∆P_L,dyn_ varied substantially according to the effect of PEEP on dynamic lung compliance (Fig. 3C). Participants were more likely to meet targets at the PEEP level associated with higher dynamic lung compliance (50% vs. 10%, median OR 33, 95% CI 5, 898) (Fig. 3D). The PEEP level resulting in higher dynamic lung compliance attenuated ∆Pes (mean difference 3 cm H_2_O, 95% CrI 1, 4 cm H_2_O) and ∆P_L,dyn_ (mean difference –3 cm H_2_O, 95% CrI –5, –1 cm H_2_O). Modifying PEEP did not affect Edi (Additional file 1: Fig. S5).

### Phase 2—Optimizing spontaneous breathing: specific effect of extracorporeal CO_2_ removal

**Fig. 4:**
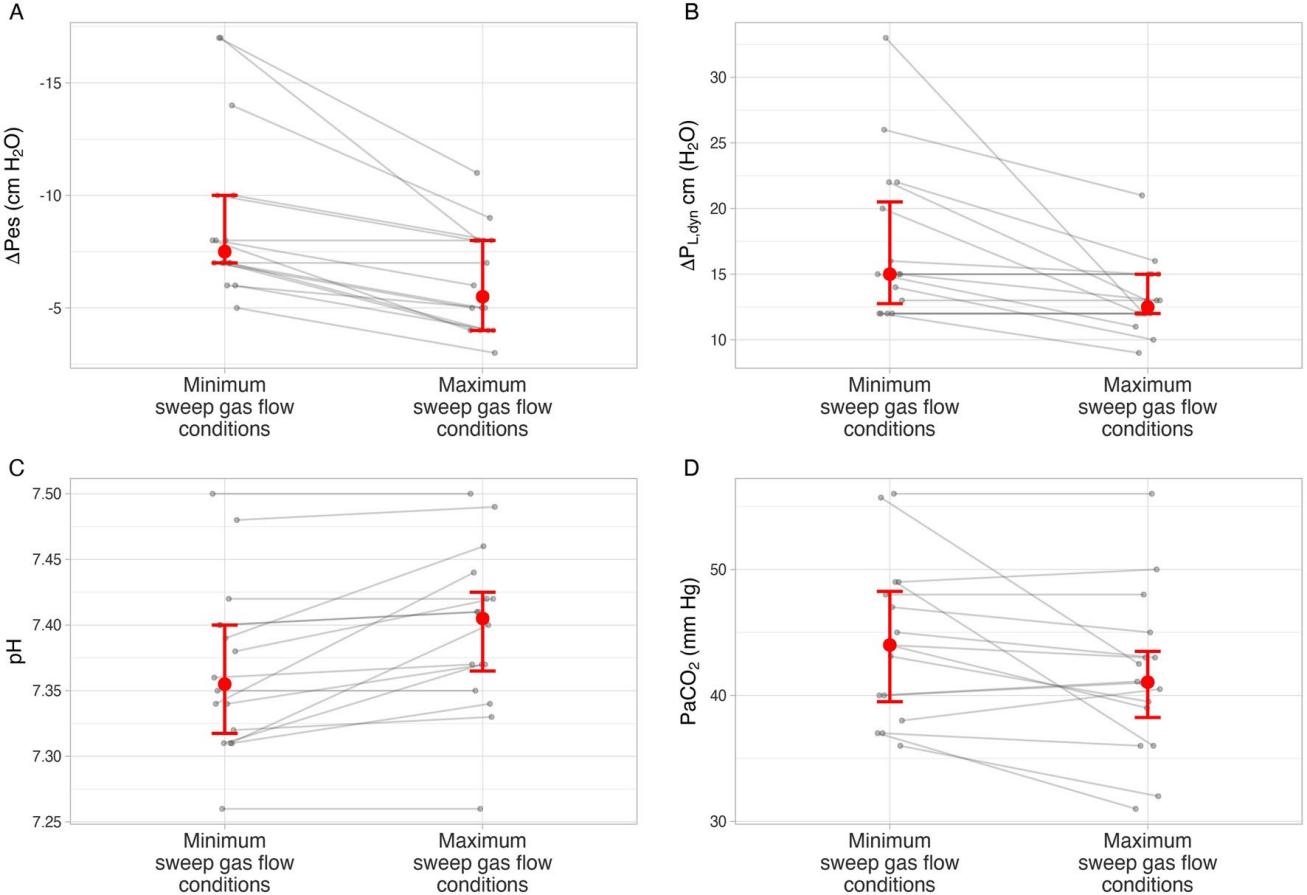
Effect of increasing sweep gas flow on ventilation, respiratory effort, and lung-distending pressure. Error bars represent 25th and 75th percentiles with median (circle). ∆Pes: esophageal pressure swing, ∆P_L,dyn_: dynamic transpulmonary driving pressure

After minimizing sweep gas flow in patients on VV-ECMO during phase 1, median (IQR) sweep gas flow was 5 (4, 5) L/min. After performing the LDP titration procedure at both PEEP levels, sweep gas flow was increased to median (IQR) 8 (7, 10) L/min. Increasing sweep gas flow attenuated ∆Pes (mean difference 5 cm H_2_O, 95% CrI 3, 7 cm H_2_O) and ∆P_L,dyn_ (mean difference –3 cm H_2_O, 95% CrI –5, 0 cm H_2_O) (Fig. 4). Patients on VV-ECMO were much more likely to meet LDP targets in comparison to patients not on VV-ECMO (88% vs. 43%, median OR 10, 95% CrI 2, 81) (Fig. 2), despite more severe impairments in respiratory mechanics and gas exchange (Additional file 1: Table S2).

### Phase 2—Optimizing spontaneous breathing: specific effect of partial neuromuscular blockade

Of the 10 patients who failed to meet LDP targets during the LDP titration procedure pha*se*, 6 (2 receiving VV-ECMO and 4 not receiving VV-ECMO) received partial neuromuscular blockade with cisatracurium (median (IQR) dose 2.5 mg (2–4 mg)). Median (min–max) SAS during this phase was 1 (1–1). Partial neuromuscular blockade attenuated ∆Pes (mean difference 6 cm H_2_O, 95% CrI 3, 10 cm H_2_O) and ∆P_L,dyn_ (mean difference –9 cm H_2_O, 95% CrI –15, –4 cm H_2_O). LDP targets were achieved in all 6 patients (Fig. 2). No respiratory acidosis, cardiopulmonary instability, hypertension, tachycardia, or distress was observed.

### Overall results of intervention

After completing the full protocol, LDP targets were achieved in 26/30 patients, including 10/14 patients not on VV-ECMO and 16/16 patients on VV-ECMO. In the remaining 4 patients who did not achieve LDP targets, permission for administration of partial neuromuscular blockade was declined by the clinical team.

## Discussion

In this physiological trial in patients with moderate to severe acute hypoxemic respiratory failure, LDP targets were safely and effectively achieved by systematically titrating ventilation and sedation, adjusting PEEP, increasing sweep gas flow (in patients on VV-ECMO), and (if needed) administering partial neuromuscular blockade. The effect of PEEP on respiratory effort varied markedly between patients, suggesting that PEEP has an important but unpredictable effect on respiratory effort in these patients and that, in order to optimize respiratory effort, the PEEP level must be individualized.

Our findings suggest that respiratory effort may often be either insufficient or excessive in patients with moderate or severe AHRF, putting them at high risk of lung or diaphragm injury. At enrolment, all patients were passively ventilated and apneic, signifying a risk of diaphragm disuse atrophy. When sedation and ventilation were initially reduced to restore respiratory drive and initiate spontaneous breathing, nearly all patients transitioned to exhibiting excessive respiratory effort and lung-distending pressure, suggesting a potential risk of patient self-inflicted lung injury or load-induced diaphragm myotrauma. The ‘brittle’ behaviour of respiratory drive and effort in these patients highlights the fundamental challenge of safe spontaneous breathing: respiratory effort must be optimized to avoid both extremes.

### Role of sedation

Sedation represents a key component of the LDP strategy as it can improve patient-ventilator synchrony, reduce respiratory effort, and prevent lung overdistention. However, if sedation is excessive, it can suppress respiratory drive [13], increase the incidence of ineffective triggering [14], and lead to diaphragm disuse atrophy [15, 16]. Because sedation scores are poorly correlated to respiratory drive and effort [17] and are insensitive to the presence of dyssynchrony, a strategy that aims to control respiratory effort must titrate sedation to the intensity of respiratory effort and not only according to traditional sedation scales, with close monitoring of patient-ventilator interaction. Further research is required to establish the optimum sedative strategy to maintain a safe spontaneous breathing pattern and to assess the trade-off with other complications of deep sedation such as prolonged immobility and delirium [18, 19].

### Role of PEEP

Higher PEEP may theoretically facilitate safe spontaneous breathing by improving respiratory mechanics, ameliorating pendelluft, and attenuating respiratory effort [9, 20]. In this trial, when changes in PEEP (either decreases or increases) were associated with improved lung mechanics, respiratory effort and lung-distending pressure were effectively attenuated and the probability of achieving LDP targets was increased. We conclude that individualized PEEP titration is likely required to optimize spontaneous breathing.

### Role of extracorporeal gas exchange

Extracorporeal CO_2_ removal reduces ventilatory demands and hence can attenuate respiratory drive [21, 22]. In this trial, receipt of VV-ECMO was associated with a much higher probability of achieving LDP targets, despite more severe physiological derangement. Increasing sweep gas flow effectively and consistently reduced respiratory effort and lung-distending pressure. Extracorporeal CO_2_ removal may therefore have an important role in facilitating safe spontaneous breathing while alleviating the need for increases in sedation to control respiratory effort.

### Role of partial neuromuscular blockade

Partial neuromuscular blockade with low doses of cisatracurium were well-tolerated and effectively attenuated elevated respiratory effort when it was refractory to other interventions. This intervention was only administered for a short period of time until LDP targets were achieved, and the feasibility and safety of maintaining partial neuromuscular blockade over hours to days remains to be established.

### Limitations

This study has several limitations. First, given the complexity of the LDP algorithm and the high acuity of the population studied in this centre, the feasibility of reproducing the results in other centres is uncertain. An experienced physician and respiratory therapist were in constant attendance at the bedside throughout the procedure. The use of a predefined algorithm to titrate ventilator settings and sedation may facilitate the reproducible evaluation of our protocol by other researchers. Second, the optimal ranges for respiratory effort and lung-distending pressure for the LDP approach remain somewhat uncertain. We employed values proposed by an international consensus group [5], but less conservative limits may still be safe and could increase the proportion of patients in whom LDP targets are achieved, as suggested in the sensitivity analysis. Third, the importance of prioritizing dynamic vs. static lung-distending pressures to prevent VILI and P-SILI is uncertain; our protocol focused on dynamic lung-distending pressure which may reflect resistive pressures of uncertain pathophysiological significance. However, dynamic pressures may more accurately reflect regional lung distention [23]. Fourth, the durability of achieving LDP targets over a more prolonged period of time (days) remains unknown. Our present goal was to describe their short-term physiological effects, but respiratory effort may vary over time [24] and ultimately more prolonged monitoring and intervention will be needed to ensure patients remain within lung and diaphragm-protective targets over prolonged periods of time. Clinical trials testing the net clinical benefit of optimizing respiratory effort for lung and diaphragm protection using a systematic approach to integrate ventilatory support and sedation are needed.

## Conclusions

Respiratory effort is frequently absent or excessive in patients with AHRF. A systematic approach to adjusting ventilation and sedation combined with partial neuromuscular blockade can achieve lung- and diaphragm-protective targets in most patients.

## Supplementary Information

**Additional file 1**. Supplemental description of methods and supplemental results.

## Data Availability

The datasets used and/or analysed during the current study are available from the corresponding author on reasonable request.

## Abbreviations

AHRF: Acute hypoxemic respiratory failure
LDP: Lung- and diaphragm-protection
PEEP: Positive end-expiratory pressure
VV-ECMO: Veno-venous extracorporeal membrane
pNMBA: Partial neuromuscular blockade
∆_PL,dyn_: Dynamic transpulmonary driving pressure
∆_Pes_: Esophageal pressure
ICU: Intensive care unit
Edi: Diaphragm electrical activity
OR: Odds ratio
CrI: Credible interval
IQR: Interquartile range
COPD: Chronic obstructive pulmonary disease
SOFA: Sequential organ failure assessment
SAS: Sedation-agitation score
*V*_T_: Tidal volume
PBW: Predicted body weight
ECLS: Extracorporeal life support
