# Supplementary material for: Strategies for lung- and diaphragm-protective ventilation in acute hypoxemic respiratory failure: a physiological trial
Source: Crit Care. 2022 Aug 29;26:259. doi: 10.1186/s13054-022-04123-9 (PMC9422941; doi:10.1186/s13054-022-04123-9)
Supplement: Supplementary file 1 — Additional file 1. Supplemental description of methods and supplemental results. [file 13054_2022_4123_MOESM1_ESM.docx]

**Supplementary Information**

**Lung- and Diaphragm-Protective Ventilation in Acute Hypoxemic Respiratory Failure: A Pilot Physiological Trial**

Jose Dianti^1,2^, Samira Fard^3^, Jenna Wong^2^, Timothy CY. Chan^4^, Lorenzo Del Sorbo^1,2^, Eddy Fan^1,2^, Marcelo B. Passos Amato^5^, John Granton^1,2^, Lisa Burry^1,6,7^, W. Darlene Reid^1,8^, Binghao Zhang^4^, Damian Ratano^1^, Shaf Keshavjee^9^, Arthur S. Slutsky^1,10^, Laurent J. Brochard^1,10^, Niall D. Ferguson^1,2,11,12,13^, Ewan C. Goligher^1,2,11^

1. Interdepartmental Division of Critical Care Medicine, University of Toronto, Toronto, Canada
2. Division of Respirology, Department of Medicine, University Health Network, Toronto, Canada
3. Department of Respiratory Therapy, University Health Network, Toronto, Canada
4. Department of Mechanical and Industrial Engineering, University of Toronto, Toronto, Canada
5. Heart Institute (Incor), Hospital das Clínicas da Faculdade de Medicina da Universidade de São Paulo, Brazil
6. Department of Pharmacy, Mount Sinai Hospital, Toronto, Canada
7. Leslie Dan Faculty of Pharmacy, University of Toronto, Toronto, Canada
8. Department of Physical Therapy, University of Toronto, Toronto, Canada
9. Department of Surgery, University of Toronto, Toronto, Toronto, Canada
10. Keenan Centre for Biomedical Research, Li Ka Shing Knowledge Institute, St. Michael’s Hospital, Toronto, Canada
11. Toronto General Hospital Research Institute, Toronto, Canada
12. Institute for Health Policy, Management, and Evaluation, University of Toronto, Toronto, Canada
13. Department of Physiology, University of Toronto, Toronto, Canada

Address for Correspondence:

Ewan C. Goligher, MD PhD

Toronto General Hospital

9-MaRS-9024

585 University Avenue

Toronto, ON M5G 2N2

(416) 340-4800 ext. 6810

ewan.goligher@uhn.ca

**Contents**

Study Population and Setting

Measurements

Study Protocol

Details on the Statistical Analysis

Changes in Ventilation and Sedation During the LDP titration procedure

**Figure S1.** Sedation minimization strategy

**Figure S2.** Algorithm for titrating inspiratory pressure and sedation to achieve lung- and diaphragm-protective targets

**Figure S3.** Flow of patients in the LANDMARK trial

**Table S1.** Mechanical ventilation and gas exchange variables at each phase of the protocol

**Figure S4.** Changes in sedation dose at each phase of the protocol

**Table S2.** Patient characteristics and ventilation variables before randomization

**Figure S5.** Effect of PEEP on diaphragm electrical activity

References

**Study Population and Setting**

The LANDMARK trial is a pilot randomized trial designed to evaluate the feasibility and safety of a novel lung and diaphragm protective (LDP) strategy in patients with acute hypoxemic respiratory failure. Randomization was performed by a computerized randomization algorithm with allocation concealment. Randomization was performed in blocks according to the three patient subgroups (mild/moderate hypoxemia, moderate/severe hypoxemia, VV-ECLS) under study.

This study was conducted in a medical-surgical intensive care unit in a tertiary academic hospital. We enrolled patients with acute hypoxemic respiratory failure who were receiving invasive mechanical ventilation. Patients were enrolled as soon as possible after admission to the unit by daily screening; however, duration of mechanical ventilation was not an exclusion criterion. The random allocation sequence and participants enrolment and assignment to intervention was performed by a research assistant (JW).

*Eligibility criteria*

*Inclusion Criteria:*

- PaO_2_:FiO_2_ ≤300 mm Hg at time of screening (or cannulation for veno-venous extracorporeal membrane oxygenation (VV ECMO))
- Oral endotracheal intubation and mechanical ventilation
- Bilateral airspace opacities on chest radiograph or chest computed tomography scan

*Exclusion Criteria:*

- Contraindication to esophageal catheterization (upper gastrointestinal tract surgery within preceding 6 weeks, bleeding esophageal/gastric varices)
- Intubated for traumatic brain injury or stroke
- Intracranial hypertension (either suspected or diagnosed by medical team)
- Anticipated liberation from mechanical ventilation within 24 hours

Patients were divided into predefined subgroups: a) patients with mild/moderate hypoxemia (PaO_2_:FiO_2_ 300-150 mm Hg), b) patients with moderate/severe hypoxemia (PaO_2_:FiO_2_ ≤150 mm Hg), and c) patients receiving extracorporeal life support with VV ECMO.

**Measurements**

*Esophageal pressure*

An esophageal catheter (NutriVent, Modena, Italy) was placed to estimate pleural pressure by measuring esophageal pressure (Pes). The catheter was connected to a dedicated monitoring system (FluxMed, Buenos Aires, Argentina) and pressure tracings were observed in real time using the FluxView software (FluxMed, Buenos Aires, Argentina). The esophageal balloon was inflated with 4 ml of air (the balloon was inflated with 6 cc to avoid folds and then 2 cc were removed). An end-expiratory occlusion maneuver was performed to quantify the difference in pressure change between the airway and the esophageal pressure during an active inspiratory effort by the patient to validate the measurement [1]. A deviation of <20% between ∆Paw and ∆Pes was considered acceptable. If this could not be achieved, in-vivo calibration with increasing filling pressures up to 8 ml was performed as suggested by Mojoli et al. [2]. Again, an end-expiratory occlusion was performed to validate the measurement. Acceptable ΔPaw/ΔPes ratios were obtained in all cases.

Airway pressure (Paw), flow, and tidal volume (V_T_) were measured by placing a pneumotachograph at the airway opening and using the FluxMed device (MBMed, Buenos Aires, Argentina).

Transpulmonary pressure (P_L_) was computed by real-time subtraction of Pes from Paw. Static ∆Paw and ∆P_L_ were measured by applying a transient end-inspiratory hold and a transient end-expiratory hold on the ventilator to measure plateau airway pressure (Pplat) and total PEEP (PEEP_tot_), and plateau esophageal pressure (Pes,ei) and end-expiratory esophageal pressure (Pes,ee), respectively.

Driving airway pressure (∆Paw) was computed as:

$$\Delta Paw= P_{plat}- {PEEP}_{tot}$$

Driving transpulmonary pressure (∆P_L_) was computed as:

$${\Delta P}_{L}= {(P}_{plat}- P_{es, ei})- {(PEEP}_{tot}- P_{es,ee})$$

Dynamic lung compliance (C_L_) was computed as:

$$C_{L,dyn}= \frac{V_{T}}{{\Delta P}_{L,dyn}}$$

Lung elastance (E_L_) was computed as:

$$E_{L}= \frac{{\Delta P}_{L}}{V_{T}}$$

Normalized E_L_ by predicted body weight (PBW) was computed to account for the difference in lung size according to height [3].

∆Pes was computed as the difference between end-expiratory Pes and the nadir of inspiratory Pes. ∆P_L,dyn_ was computed as the difference between peak airway pressure and ∆Pes.

*Electrical diaphragm activity*

Electrical diaphragm activity (Edi) was measured by an esophageal electromyogram catheter (Maquet, Solna, Sweden) connected to the Servo-U ventilator (Maquet, Solna, Sweden). The reported Edi represents the mean value of Edi peak in a 30 second period.

**Study Protocol**

We employed a stepwise algorithm for systematically titrating ventilation and sedation to achieve LDP targets. The goal of this strategy is to prevent both disuse-mediated and load-induced diaphragm injury while also preventing excess global and regional mechanical stress and strain in the injured lung. The algorithm relied on esophageal manometry for direct monitoring of ∆Pes and ∆P_L,dyn_. Targets for lung and diaphragm protection were selected based on a recent those suggested in a recent statement by a panel of experts [4]. Inspiratory pressure, PEEP, sedation, and sweep gas flow (in patients receiving VV ECMO) were systematically titrated to achieve LDP. All patients were in a pressure limited mode (either PC-CMV or PC-CSV) after enrolment. Cycling criteria and rise time were adjusted to minimize patient-ventilator dyssynchrony before commencing the protocol. A propofol-based sedation strategy was used. Titrations were conducted by a team of experienced clinicians comprised of a respiratory therapist and a physician (SF and JD). Tidal volumes were kept below 10 ml/kg predicted body weight at all times and maximal propofol dose was 80 µg/kg/min.

Clinical management of hypoxemic respiratory failure in our ICU follows current recommendations for the management of patients with moderate/severe ARDS, including recommendation for patients receiving VV-ECMO. Specifically, these include low tidal volume ventilation with plateau pressure limitation and higher PEEP for patients not on ECMO, and inspiratory pressure and PEEP of 10 cm H_2_O for patients on VV-ECMO.

The protocol proceeded in two mandatory phases (Figure 1 in the main manuscript). Study participants on VV ECMO underwent a third step in the *LDP titration procedure phase* where sweep gas flow was increased (maximum sweep gas flow step). In participants who did not meet LDP targets, partial neuromuscular blockade was applied to reduce the magnitude of the inspiratory effort.

*Phase 1: Initiate spontaneous breathing.* Patients were enrolled as early as possible since admission to our intensive care unit (ICU) by daily screening by the research team (JD and/or JW). After enrolment, sedation and respiratory rate were adjusted using a standardized procedure (Figure S1) to facilitate the initiation of patient respiratory effort until the ventilator was continuously triggered by the patient on every breath. In patients on VV-ECMO, sweep gas flow was also reduced to the minimum level required to maintain pH >7.3, plateau pressure <30 cm H_2_O, and respiratory rate <35 breaths per minute. All patients were ventilated in a pressure-targeted mode but ventilator settings during this phase were otherwise managed by the clinician in charge of the patient following usual clinical practice. ∆Pes and ∆P_L,dyn_ were not monitored by the clinician in charge.

*Phase 2: Optimize spontaneous breathing to achieve LDP targets.* Once patients were continuously triggering the ventilator with minimal or no dyssynchrony and SpO_2_ was >88%, inspiratory pressure and sedation were systematically titrated to modulate respiratory effort (quantified by ∆Pes) and lung-distending pressure (∆P_L,dyn_) to achieve LDP targets while maintaining adequate ventilation (pH >7.25) (Figure 1 in the main manuscript). The target range for respiratory effort (∆Pes –3 to –8 cm H_2_O) was selected to avoid both disuse atrophy and load-induced diaphragm injury [5–7]. The target for lung-distending pressure (∆P_L,dyn_ ≤15 cm H_2_O) was selected based on acceptable levels of dynamic lung stress in experimental models and based on data on the accepted upper limit for static driving pressure in patients with ARDS [3, 8]. The rationale for these targets was developed by consensus and has previously been described in detail [4].

To test the hypothesis that higher PEEP increased the probability of achieving LDP targets, patients were randomly assigned to lower PEEP (PEEP of 8 cm H_2_O or the lowest PEEP level required to maintain FiO_2_ ≤90% and SpO_2_ ≥90%) or higher PEEP (PEEP sufficient to maintain end-expiratory P_L_ between 2-3 cm H_2_O).

Inspiratory pressure and sedation were then adjusted according to a pre-defined stepwise algorithm for systematically titrating ventilation and sedation to achieve LDP targets (Figure S2). Briefly, if respiratory effort was elevated and pH was lower than 7.35, inspiratory pressure was increased to reduce respiratory drive and effort. If effort was elevated and acid-base homeostasis was within normal limits, sedation was increased to reduce respiratory drive and effort. If respiratory effort was low and pH was within normal limits, sedation was decreased to increase respiratory drive and effort. If respiratory effort was acceptable and lung-distending pressure was excessive, inspiratory pressure was decreased. The magnitude of the changes in inspiratory pressure and sedation at each titration step was determined by the physician conducting the protocol at the bedside (JD). Up to three cycles through the algorithm were performed to determine whether all LDP targets as specified above could be achieved simultaneously.

A 10-minute stabilization period at this PEEP level was required before continuing to the next step. After the titration procedure was completed at the first randomly assigned PEEP level, patients crossed over to the opposite PEEP level and, after a 10-minute stabilization period, the titration algorithm was repeated to determine whether LDP targets could be achieved at the alternate PEEP level. The procedure was considered successful if all LDP targets were met at the end of the 10-minute stabilization period.

Patients receiving VV-ECMO underwent a third step where sweep gas flow was increased to a maximum of 10 L/min to enhance extracorporeal CO_2_ removal. After a 10-minute stabilization period the LDP titration procedure was repeated.

Finally, in patients in whom LDP targets could not be achieved at the end of the above procedures because of persistent excessive respiratory effort, partial neuromuscular blockade was administered if permitted by the clinical team. Bolus doses of 0.5-1 mg cisatracurium were administered every 3 minutes until ∆Pes was above –8 cm H_2_O. ∆P_L,dyn_ and ∆Pes were then recorded.

Patients were continuously monitored for respiratory deterioration (development of severe respiratory acidosis with pH < 7.2; severe hypoxemia with FiO_2_ increased by more than 0.2) or hemodynamic deterioration (vasopressor requirements increase by 25% or lactate increase by 4 mM).

Serious adverse events were monitored and documented as: (1) severe respiratory acidosis (pH < 7.25), (2) prolonged severe hypoxemia (oxygen saturation < 85% for > 2 minutes) during the LDP algorithm titration, (3) severe metabolic acidosis (lactate > 4 mM or increase in lactate by > 4 mM), (4) hemodynamic instability, (5) agitation or anxiety, (6) respiratory distress (evidenced by the use of accessory respiratory muscles and/or paradoxical breathing) or (7) severe patient-ventilator dyssynchrony.

**Details on the Statistical Analysis**

There are no previous data on the feasibility of achieving LDP in patients with acute hypoxemic respiratory failure to inform sample size calculation. Hypothesizing that 80% of the patients would meet LDP targets at any point of the LDP titration procedure (including the maximum sweep gas flow step), we estimated a sample size of 30 subjects would permit an estimation of the proportion of patients in which LPD targets were achieved with confidence intervals of ±16%.

For the primary outcome, we report the proportion of patients in whom LDP targets were achieved at the end of the *LDP titration procedure phase* under any condition (lower or higher PEEP, and in patients on VV ECMO, minimum or maximum sweep gas flow) along with their respective 95% confidence intervals (CI), estimated using the binomial distribution. We also report this proportion and its 95% confidence interval (CI) at enrolment and at the end of the *sedation minimization phase* (prior to any adjustment of the LDP titration algorithm). Continuous variables are described as mean (±SD) or median (IQR) depending on their distribution. Categorical variables are described as counts (%).

To evaluate the effect of the intervention on the probability of achieving LDP targets we used a Bayesian generalized mixed effects model clustered by patient to derive a) effect estimates with their corresponding 95% credible intervals, and b) the posterior probability of an effect. Specifically, we used the 50^th^, 2.5^th^, and 97.5^th^ percentiles of the posterior distribution to derive the median and 95% credible intervals (CrI) for the odds ratio, respectively. The posterior probability of an effect was estimated as the posterior probability of odds ratio > 1. The model was fitted using the binomial distribution and logit-link function, with 4 chains and 8000 iterations (4000 iterations for the warm-up period). We used non-informative priors given the lack of data regarding the feasibility of achieving LDP targets. A similar model was fit to compare the probability of meeting LDP targets between patients who were or were not receiving VV ECMO. Finally, to test whether receiving VV ECMO modifies the probability of achieving LDP targets, a similar model was fit adding and interaction term between study phase (before *LDP titration procedure phase* and after) and being on VV ECMO.

To evaluate the effect of the different PEEP levels and of the different sweep gas flow levels on inspiratory effort (quantified by ∆Pes), we fitted Bayesian generalized mixed effects models clustered by patient. To isolate the effect of PEEP on these endpoints, the values obtained at the end of *LDP titration procedure* *phase* at the first PEEP level were compared to the values obtained following PEEP adjustment at the alternate PEEP level (following a 10-minute stabilization, but before any other adjustments to ventilation or sedation). Similarly, these endpoints were compared at minimum and maximum sweep gas flow conditions in patients receiving VV ECMO. The normal distribution and non-informative priors were used to estimate the posterior distribution. Similarly, we used the 50^th^, 2.5^th^, and 97.5^th^ percentiles of the posterior distribution to derive the median and 95% credible intervals (CrI) for the odds ratio, respectively. The posterior probability of an effect was estimated as the posterior probability of odds ratio > 1. Finally, to assess whether the effect of PEEP on respiratory effort was modified by each patient’s potential for lung recruitment, the model evaluating the association between PEEP and ∆Pes specified an interaction term with lung recruitability (defined as an improvement in lung compliance at higher PEEP, see the assessment of *lung recruitment section* for rationale).All statistical analyses were performed using R v1.4.1717 (R Foundation for Statistical Computing, Vienna, Austria, https://www.R-project.org/). Package {rstanarm} was used to fit all the Bayesian models [9].

**Changes in Ventilation and Sedation During the LDP titration procedure**

Inspiratory pressure was increased in 9/30 patients (31%) and decreased in 9/30 patients (31%). The propofol infusion rate was increased in 17/30 (57%) and decreased in 2/30 patients (7%). The fentanyl infusion rate was increased in 2/30 (7%) and decreased in 3/30 patients (10%). Propofol and fentanyl infusion rates at each step in the protocol are shown in Figure S4.

**F****igure S1.** Sedation minimization strategy.

SAS: sedation-agitation score, IV: intravenous.

**Figure S2.** Algorithm for titrating inspiratory pressure and sedation to achieve lung- and diaphragm-protective targets.

^*^Units for ∆Pes and ∆P_L,dyn_ are in cm H_2_O.

∆Pes: esophageal pressure swing, ∆P_L,dyn_: dynamic driving transpulmonary pressure, Pvent: set inspiratory pressure, 3^rd^ loop: third attempt to modify inspiratory pressure/sedation. If lung- and diaphragm-protective targets of ∆Pes 3-8 cm H_2_O and ∆P_L,dyn_ ≤ 15 cm H_2_O were not achieved after this 3^rd^ iteration, the algorithm was considered as failed.

**Figure S3.** Flow of patients in the LANDMARK trial.

GI: gastrointestinal, MV: mechanical ventilation, IQR: interquartile range, LDP: lung and diaphragm protection.

**Table S1.** Mechanical ventilation and gas exchange variables at each phase of the protocol.

|  | **After initiation of spontaneous breathing (n= 30)** | **After LDP titration** | | **After increasing sweep gas flow (n= 16)** | **After addition of pNMBA**  **(n= 6)** |
| --- | --- | --- | --- | --- | --- |
|  |  | **Higher PEEP**  **(n= 29)** | **Lower PEEP**  **(n= 29)** |  |  |
| RF (b/min), median (IQR)  Total  Success*  Failure† | 25 (20, 34)  24 (21, 30)  27 (20, 36) | 22 (16, 28)  23 (18, 28)  22 (12, 31) | 22 (1, 30)  22 (18, 30)  22 (11, 31) | 23 (24, 30)  23 (16, 28)  23 (18, 30) | 21 (13, 30)  n/a |
| V_T_ (ml/kg PBW), median (IQR)  Total  Success  Failure | 6.0 (4.7, 7.6)  5.5 (4.1, 7.3)  6.9 (5.7, 7.8) | 5.5 (4.0, 6.8)  4.7 (3.4, 6.3)  6.8 (5.7, 8.0) | 6.2 (4.0, 7.5)  5.2 (3.4, 6.2)  7.4 (6.4, 9.2) | 3.6 (2.2, 4.6)  3.5 (2.0, 4.0)  5.1 (4.3, 6.0) | 7 (7, 9)  n/a |
| PEEP (cm H_2_O), median (IQR)  Total  Success  Failure | 10 (8, 14)  10 (10, 14)  10 (8, 14) | 14 (13, 16)  14 (14, 16)  13 (12, 16) | 8 (8, 8)  8 (8, 8)  8 (8, 8) | 11 (8, 14)  9 (8, 14)  16 (15, 18) | 11 (8, 14)  n/a |
| ∆P (cm H_2_O), median (IQR)  Total  Success  Failure | 17 (12, 19)  15 (12, 18)  17 (15, 23) | 15 (12, 16)  13 (11, 15)  19 (15, 25) | 15 (13, 17)  14 (11, 17)  18 (16, 26) | 13 (10, 14)  13 (10, 14)  14 (13, 16) | 13 (11, 17)  n/a |
| Inspiratory pressure (cm H_2_O), median (IQR)  Total  Success  Failure | 10 (10, 14)  10 (10, 12)  11 (8, 14) | 12 (10, 15)  10 (8, 14)  15 (12, 16) | 11 (10, 14)  10 (9, 14)  13 (10, 15) | 9 (8, 14)  10 (8, 14)  9 (8, 9) | 9 (7, 10)  n/a |
| ∆Pes (cm H_2_O), median (IQR)  Total  Success  Failure | -12 (-8, -19)  -10 (-7, -13)  -22 (-14, -26) | -8 (-7, -12)  -7 (-7, -8)  -17 (-13, -20) | -9 (7, -15)  -7 (-7, -10)  -14 (-13, -15) | -5 (-4, -8)  -5 (-4, -8)  -10 (-9, -10) | -7 (-8, -6)  n/a |
| ∆P_L,dyn_ (cm H_2_O), median (IQR)  Total  Success  Failure | 19 (15-26)  16 (14, 18)  28 (22, 33) | 15 (14, 21)  14 (13, 15)  24 (21, 31) | 17 (14, 22)  15 (13, 20)  20 (19, 28) | 12 (12, 15)  12 (12, 13)  18 (17, 20) | 14 (12, 14)  n/a |
| Pocc (cm H_2_O), median (IQR)  Total  Success  Failure | 16 (12, 21)  14 (11, 16)  31 (21, 36) | 14 (10, 17)  12 (9, 15)  23 (13, 29) | 15 (9, 20)  13 (8, 17)  23 (11, 26) | 9 (6, 13)  9 (6, 12)  15 (15, 15) | 11 (7, 15)  n/a |
| P0.1 (cm H_2_O), median (IQR)  Total  Success  Failure | 3.3 (1.8, 5.9)  3.3 (1.8, 4.2)  8.0 (1.9, 9.1) | 3.1 (2.0, 4.3)  2.7 (2.1, 3.9)  4.1 (1.8, 5.6) | 2.3 (1.3, 4.3)  2.2 (1.2, 3.5)  3.4 (2.0, 5.2) | 1.4 (1.2, 2.6)  1.3 (1.2, 2.2)  2.6 (2.3, 2.9) | 3.3 (1.4, 5.8)  n/a |
| PaO_2_ (mm Hg), median (IQR)  Total  Success  Failure | 76 (67, 86)  74 (67, 84)  79 (74, 98) | 81 (65, 95)  78 (65, 93)  86 (74, 98) | 77 (66, 90)  77 (68, 91)  79 (62, 90) | 79 (63, 84)  79 (67, 83)  71 (63, 80) | 81 (75, 84)  n/a |
| PaCO_2_ (mm Hg), median (IQR)  Total  Success  Failure | 43 (41, 53)  42 (41, 49)  47 (42, 66) | 47 (42, 56)  47 (43, 50)  46 (40, 60) | 44 (40, 53)  44 (40, 53)  44 (39, 57) | 41 (38, 43)  41 (37, 44)  42 (41, 42 | 46 (43, 49)  n/a |
| pH, median (IQR)  Total  Success  Failure | 7.36 (7.31, 7.44)  7.37 (7.32, 7.43)  7.35 (7.30, 7.45) | 7.35 (7.29, 7.41)  7.35 (7.31, 7.40)  7.30 (7.23, 7.41) | 7.35 (7.31, 7.43)  7.36 (7.34, 7.42)  7.31 (7.28, 7.44) | 7.40 (7.36, 7.42)  7.40 (7.37, 7.43)  7.37 (7.35, 7.39) | 7.36 (7.29, 7.41)  n/a |

*Success represents patients who met LPD targets at any point during the LDP titration.

†Failure represents patients who never met LDP targets.

RF: respiratory frequency. V_T_: tidal volume, PBW: predicted body weight, PEEP: positive end-expiratory pressure, ∆P: airway driving pressure, ∆Pes: esophageal pressure swing, ∆P_L,dyn_: dynamic driving transpulmonary pressure, Pocc: airway end-expiratory occlusion pressure, P0.1: inspiratory pressure in the first 100 milliseconds, pNMBA: partial neuromuscular blocking agent.

**Figure S4.** Changes in sedation dose at each phase of the protocol.

Change in the dose of propofol (**A**) and fentanyl (**B**) throughout the protocol. The posterior probability of a difference in mean fentanyl dose larger than 10 µg/kg/min between patients who met and those who did not meet LDP targets was 72% at enrolment, 99% after initiating spontaneous breathing, and 99% after the LDP titration at both PEEP levels. Posterior probability of a difference in mean propofol larger than 10 µg/kg/min was less than 50% across all phases.

LDP: lung and diaphragm protection, PEEP: positive end-expiratory pressure.

**Table S2.** Patient characteristics and ventilation variables before randomization between patients receiving VV-ECMO and not receiving VV-ECMO (before partial neuromuscular blockade phase).

|  | **On VV ECMO (n= 16)** | | | | **Not on VV ECMO (n= 14)** | | | |
| --- | --- | --- | --- | --- | --- | --- | --- | --- |
|  | **Met LDP targets (n= 12)** | **Did not meet LDP targets (n= 4)** | **SMD** | **p** | **Met LDP targets (n= 6)** | **Did not meet LDP targets (n= 8)** | **SMD** | **p** |
| Age, median (IQR) | 49 (31, 55) | 52 (45, 53) | 0.26 | 0.81 | 61 (57, 65) | 57 (52, 60) | 0.94 | 0.19 |
| Female sex, n (%) | 5 (41) | 1 (25) | 0.36 | 0.55 | 3 (50) | 1 (12) | 0.88 | 0.12 |
| APACHE II, median (IQR) | 19 (18, 21) | 21 (18, 25) | 0.41 | 0.85 | 25 (20, 27) | 24 (20, 30) | 0.35 | 0.63 |
| SOFA, median (IQR) | 10 (10, 11) | 11 (10, 13) | 0.60 | 0.40 | 10 (10, 11) | 11 (11, 11) | 0.22 | 0.50 |
| ARDS severity  Mild  Moderate  Severe | 0 (0)  0 (0)  12 (100) | 0 (0)  0 (0)  4 (100) | 0 | 0.05 | 0 (0)  4 (67)  2 (33) | 0 (0)  3 (37)  5 (63) | 0.61 | 0.28 |
| SAS, median (IQR) | 1 (1, 1) | 2 (1, 2) | 1.11 | 0.02 | 1 (1, 2) | 1 (1, 2) | 0.1 | 0.88 |
| Comorbidities, n (%)  Asthma  COPD  Interstitial lung disease  Diabetes  Chronic kidney disease  Prior lung transplant | 2 (17)  1 (8)  1 (8)  3 (25)  1 (8)  0 (0) | 0 (0)  0 (0)  0 (0)  1 (25)  0 (0)  0 (0) | 0.63  0.43  0.43  0  0.43  0 | 0.38  0.55  0.55  1.00  0.55  0.56 | 0 (0)  3 (50)  0 (0)  2 (33)  0 (0)  0 (0) | 1 (12)  1 (12)  1 (12)  2 (25)  0 (0)  1 (12) | 0.53  0.88  0.53  0.18  0  0.53 | 0.37  0.12  0.53  0.73  0.59  0.30 |
| Cause of respiratory failure, n (%)  Bacterial pneumonia  Fungal pneumonia  Influenza pneumonia  Covid-19 pneumonia | 4 (34)  1 (8)  1 (8)  6 (50) | 1 (25)  0 (0)  1 (25)  2 (50) | 0.63 | 0.79 | 1 (17)  0 (0)  0 (0)  5 (83) | 7 (88)  0 (0)  0 (0)  1 (12) | 2 | 0.01 |
| PaO_2:_FiO_2_, median (IQR)^*^ | 168 (106, 187) | 161 (131, 189) | 0.14 | 0.71 | 199 (190, 225) | 177 (120, 211) | 0.25 | 0.37 |
| Ventilatory ratio, median (IQR)† | 1.6 (1.2, 1.8) | 1.3 (1.3, 1.7) | 0.01 | 0.62 | 1.6 (1.5, 1.9) | 2.4 (2.0, 2.8) | 0.94 | 0.09 |
| ECLS flow (L/min) | 5 (4, 5) | 5 (4, 5) | 0.24 | 0.54 | n/a | n/a | n/a | n/a |
| Sweep gas flow (L/min) | 5 (4, 5) | 5 (4, 8) | 0.55 | 0.68 | n/a | n/a | n/a | n/a |
| V_T_ (ml/kg PBW), median (IQR) | 5 (3, 6) | 6 (5, 7) | 0.44 | 0.33 | 8 (7, 9) | 7 (5, 8) | 0.58 | 0.30 |
| Mode of ventilation, n (%)  Pressure support ventilation  Assist-control pressure ventilation | 11 (92)  1 (8) | 4 (100)  - | 0.43  n/a | 1  n/a | 6 (100)  - | 5 (63)  3 (37) | 1.1  n/a | 0.30  n/a |
| PEEP (cm H_2_O), median (IQR) | 11 (10, 15) | 15 (13, 15) | 0.42 | 0.34 | 8 (8, 9) | 9 (7, 10) | 0.02 | 0.89 |
| ∆P (cm H_2_O), median (IQR) | 16 (14, 17) | 14 (12, 20) | 0.16 | 0.77 | 15 (12, 19) | 18 (16, 20) | 0.71 | 0.36 |
| Adjusted respiratory system elastance (cm H_2_O / ml/kg PBW), median (IQR) | 3.5 (2.5, 5.4) | 2.0 (1.9, 3.0) | 0.89 | 0.25 | 1.9 (1.5, 2.1) | 2.6 (1.8, 5.0) | 0.96 | 0.20 |
| P0.1 (cm H_2_O), median (IQR) | 3.4 (3.0, 4.0) | 3.7 (3.1, 5.0) | 0.21 | 0.63 | 1.3 (1.2, 3.9) | 8.1 (1.7, 10.2) | 0.99 | 0.14 |
| Pocc (cm H_2_O), median (IQR) | 14 (11, 17) | 22 (17, 27) | 0.89 | 0.13 | 14 (10, 15) | 31 (19, 37) | 2.01 | 0.01 |

*Values of PaO_2_/FiO_2_ are modified by VV ECMO and may not be representative of lung function in patients receiving VV-ECMO at enrolment.

†Values of ventilatory ratio are modified by VV ECMO and may not be representative of lung function.

‡Standardized mean difference (SMD) cannot be calculated for more than two comparisons.

∆P: airway driving pressure, VV ECMO: veno-venous extracorporeal membrane oxygenation, LDP: lung and diaphragm protection, SMD: standardized mean difference, IQR: interquartile range, COPD: chronic obstructive pulmonary disease, V_T_: tidal volume, PEEP: positive end-expiratory pressure, PBW: predicted body weight.

**Figure S5.** Effect of PEEP on diaphragm electrical activity.

There was no difference in electrical diaphragm activity (Edi) between higher or lower PEEP (**A**), or the PEEP levels associated with worst and best dynamic lung compliance (**B**). The posterior probability of a difference in Edi larger than 1 µV was 20% for the lower vs. higher PEEP comparison, and 55% for the worst vs. best dynamic lung compliance comparison.

PEEP: positive end-expiratory pressure, Edi: electrical diaphragm activity.

**References**

1. Baydur A, Behrakis PK, Zin WA, et al (2015) A Simple Method for Assessing the Validity of the Esophageal Balloon Technique1–2. Am Rev Respir Dis 126:788–91. https://doi.org/10.1164/arrd.1982.126.5.788

2. Mojoli F, Torriglia F, Orlando A, et al (2018) Technical aspects of bedside respiratory monitoring of transpulmonary pressure. Ann Transl Medicine 6:377–377. https://doi.org/10.21037/atm.2018.08.37

3. Goligher EC, Costa ELV, Yarnell CJ, et al (2021) Effect of Lowering Vt on Mortality in Acute Respiratory Distress Syndrome Varies with Respiratory System Elastance. Am J Resp Crit Care 203:1378–1385. https://doi.org/10.1164/rccm.202009-3536oc

4. Goligher EC, Dres M, Patel BK, et al (2020) Lung and Diaphragm-Protective Ventilation. Am J Resp Crit Care. https://doi.org/10.1164/rccm.202003-0655cp

5. Zocchi L, Fitting JW, Majani U, et al (1993) Effect of Pressure and Timing of Contraction on Human Rib Cage Muscle Fatigue. Am Rev Respir Dis 147:857–864. https://doi.org/10.1164/ajrccm/147.4.857

6. Jubran A, Grant BJB, Laghi F, et al (2005) Weaning Prediction. Am J Resp Crit Care 171:1252–1259. https://doi.org/10.1164/rccm.200503-356oc

7. Reynolds SC, Meyyappan R, Thakkar V, et al (2016) Mitigation of Ventilator-induced Diaphragm Atrophy by Transvenous Phrenic Nerve Stimulation. Am J Resp Crit Care 195:339–348. https://doi.org/10.1164/rccm.201502-0363oc

8. Amato MBP, Meade MO, Slutsky AS, et al (2015) Driving Pressure and Survival in the Acute Respiratory Distress Syndrome. New Engl J Medicine 372:747–755. https://doi.org/10.1056/nejmsa1410639

9. B G, J G, I A, S. B (2020) rstanarm: Bayesian applied regression modeling via Stan. R package version 2.21.1. https://mc-stan.org/rstanarm
